# Intraoperative hypothermia in the neonate population: risk factors, outcomes, and typical patterns

**DOI:** 10.1007/s10877-022-00863-9

**Published:** 2022-04-22

**Authors:** Man-Qing Zhang, Peng-Dan Ying, Yu-Jia Wang, Jia-lian Zhao, Jin-Jin Huang, Fang-Qi Gong

**Affiliations:** 1 Department of Anaesthesiology, The Children’s Hospital, Zhejiang University School of Medicine, National Clinical Research Center for Child Health, Hangzhou, China; 2 Department of Cardiology, The Children’s Hospital, Zhejiang University School of Medicine, National Clinical Research Center for Child Health, No. 3333 Binsheng Road, Hangzhou, 310051 People’s Republic of China

**Keywords:** Neonates, Surgery, Hypothermia, Risk factors, Typical pattern

## Abstract

The risk factors, outcomes, and typical patterns of intraoperative hypothermia were studied in neonates to better guide the application of insulation measures in the operating room. This retrospective study enrolled 401 neonates undergoing surgery under general anaesthesia with tracheal intubation, including abdominal surgery, thoracic surgery, brain surgery, and others. The study collected basic characteristics, such as age, sex, weight, birth weight, gestational week, primary diagnosis and American Society of Anaesthesiologists (ASA) grade. Perioperative data included preoperative body temperature, length of hospital stay, length of intensive care unit (ICU) stay, intubation time, postoperative bleeding, postoperative pneumonia, postoperative death, and total cost of hospitalization. Intraoperative data included surgical procedures, anaesthesia duration, operation duration, blood transfusion, fluid or albumin infusion, and application of vasoactive drugs. The incidence of intraoperative hypothermia (< 36 °C) was 81.05%. Compared to normothermic patients, gestational week (OR 0.717; 95% CI 0.577–0.890; P = 0.003), preoperative temperature (OR 0.228; 95% CI 0.091–0.571; P = 0.002), duration of anaesthesia (OR 1.052; 95% CI 1.027–1.077; P < 0.001), and type of surgery (OR 2.725; 95% CI 1.292–5.747; P = 0.008) were associated with the risk of intraoperative hypothermia. Patients with hypothermia had longer length of ICU stay (P = 0.001), longer length of hospital stay (P < 0.001), and higher hospital costs (P < 0.001). But there were no association between clinical outcomes and intraoperative hypothermia in the multivariable regression adjusted analysis. The lowest point of intraoperative body temperature was approximately 1 h 30 min. Then, the body temperature of patients successively entered a short plateau phase and a period of slow ascent. The greatest decrease in body temperatures occurred in preterm babies and neonates with preoperative hypothermia. The lowest core temperatures that occurred in neonates with preoperative hypothermia was lower than 35 °C. This study shows that there is a high incidence of intraoperative hypothermia in the neonate population. The intraoperative body temperature of neonates dropped to the lowest point in 1–1.5 h. The greatest decrease in core temperatures occurred in preterm babies and neonates with lower preoperative temperature.

## Introduction

Inadvertent intraoperative hypothermia is a frequently preventable complication with several adverse consequences, including increased intraoperative blood loss and a need for transfusions [1], prolonged length of recovery room and hospital stays [2], cold-induced coagulation dysfunction [3], postoperative infection, and more [4, 5]. Its avoidance has been recommended and incorporated into various clinical guidelines [6, 7]. General anaesthetics greatly increase the risk for intraoperative hypothermia [8]. Volatile anaesthetics such as isoflurane and sevoflurane [9] and intravenous anaesthetics such as propofol [10] and opioids [11] all substantially impair thermoregulatory control. Consequently, unwarmed anesthetized patients become hypothermic. Hypothermia develops with a characteristic pattern in adults administered general anaesthesia. Core body temperature decreases rapidly in the first hour. This initial hypothermia is followed by a 2- or 3-h slower decrease. Finally, patients enter a plateau phase [12]. Thermoregulation is well developed at birth, and premature infants regulate temperature better than might be expected [13]. Thermoregulatory response thresholds are well preserved in infants and children undergoing surgery under general anaesthesia [14]. However, the small thermal mass and high surface-area-to-weight ratio might make infants more susceptible to environmental perturbations than adults. The core temperatures in neonates are less stable [8]. Current studies on the characteristics associated with intraoperative hypothermia in neonates are limited. Our study aimed to determine the risk factors, outcomes, and typical patterns of intraoperative hypothermia in neonates to better guide the application of insulation measures in the operating room. This is particularly important in developing countries where insulation equipment is insufficient.

## Methods

### Patients and ethics

This retrospective study was carried out at the Children’s Hospital of Zhejiang University (Hangzhou, China) after being approved by the Ethics Committee (2020-IRB-105), and the study was registered in the Chinese Clinical Trial Registry (number: ChiCTR1800018863) prospectively before the beginning of data collection. A total of 401 neonates who underwent surgery under general anaesthesia from 1 October 2018 to 15 January 2020 were enrolled. Their basic characteristics and perioperative data were collected and analysed.

### Inclusion and exclusion criteria

The inclusion criteria were as follows: neonates undergoing surgery under general anaesthesia for tracheal intubation; the duration of operation was greater than 30 min; and temperature monitoring was performed during the operation.

The exclusion criteria were as follows: fever (body temperature > 38.0 °C) due to infection or caused by other reasons within 3 days before surgery; thermoregulation abnormalities such as malignant hyperthermia, neuroleptic malignant syndrome; cardiac surgery, and conditions requiring therapeutic hypothermia; endocrine disease affecting body temperature (hyperthyroidism, hypothyroidism, etc.); and perioperative use of drugs that affect body temperature, such as tyenol.

### Anaesthesia

Routine monitoring, such as haemoglobin oxygen saturation (SpO2), electrocardiogram (ECG), noninvasive blood pressure (NIBP) or invasive blood pressure (IBP), was carried out after entering the operating room. The regimens of general anaesthesia used were mostly midazolam (0.1 mg/kg), fentanyl (3–5 µg/kg), and rocuronium (0.6–1 mg/kg) as induction and sevoflurane (1–2 vol%) for maintenance.

### Core temperature measurement

The operating room temperature is set at 26–27 ℃. All patients were warmed passively by preheated disinfectant, covering sheets, surgical draping, or warm blankets and were warmed actively by air heaters, infusion warming instruments, or radiant beds. The oesophageal temperature was monitored after anaesthesia induction or intubation. The primary outcome was inadvertent intraoperative hypothermia, defined as core temperature < 36 °C at any time during the intraoperative period. Body temperature was recorded via an oesophageal probe every 5 min during surgery.

### Data collection

Basic characteristics, such as age, sex, weight, birth weight, gestational week, primary diagnosis, preoperative body temperature, and American Society of Anaesthesiologists (ASA) grade, were collected. Intraoperative factors included operative procedures, anaesthesia duration, operation duration, blood transfusion, plasma transfusion, fluid infusion, albumin infusion, and application of vasoactive drugs. Perioperative factors were the length of hospital stay, length of intensive care unit (ICU) stay, intubation time, postoperative bleeding, postoperative pneumonia, postoperative death, and total cost of hospitalization. The data of intraoperative factors and intraoperative body temperature were obtained from the electronic anaesthesia system (Medicalsystem, Suzhou China), and other data were obtained from the electronic medical record system (Ewell, Hangzhou China).

### Statistical analyses

All data were statistically processed using SPSS 22.0. Measurement data were tested for normality. Measurement data conforming to a normal distribution were expressed as the mean ± standard deviation (mean ± SD). The comparison between the two groups was performed by the 2-independent sample t test.

Measurement data that did not conform to the normal distribution were presented as the median and interquartile range [M(Q)], and the comparison between groups was performed by the Mann–Whitney U test or Kruskal–Wallis H test. Count data were presented as n (%), and the χ2 test was used for comparison between groups. Multivariate logistic regression was used to analyse the risk factors and outcomes for hypothermia during the operation. Significant level α = 0.05.

## Results

### Incidence

**Fig. 1:**
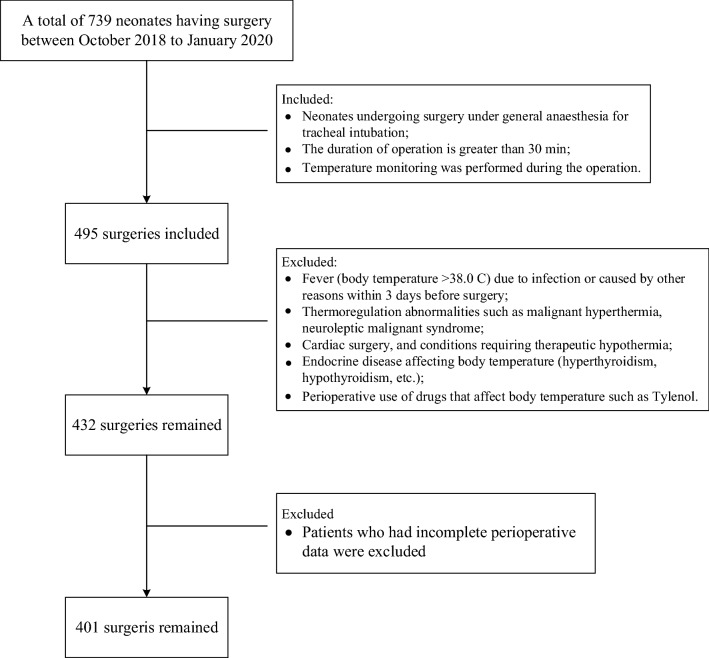
Flowchart of the study. Flowchart summarising the steps used for patient selection

Data were collected from 401 neonates between 1 October 2018 and 15 January 2020 (Fig. 1). The study procedures were well tolerated. Intraoperative hypothermia (< 36 °C) occurred in 325 cases (81.05%) in this population, and 20.45% of neonates experienced intraoperative temperature below 35 ℃. Table 1 lists the surgery category and operative procedures performed in neonates. All patients underwent invasive surgeries proven to be associated with intraoperative hypothermia.

**Table 1:** List of surgery categories and operative procedures performed in neonates

Abdominal surgery (n = 291, 72.57%)
Exploratory laparotomy with or without intestinal resection
Ladd’s procedure
Duodenal atresia or web repair
Annular pancreas correction
Pena operation
Anorectal repair
Merkel diverticulectomy
Pyloromyotomy
Omphalocele operation
Enterostomy
Hirschsprung surgery
Thoracic surgery (n = 52, 12.97%)
Tracheoesophageal fistula/oesophageal atresia repair
Diaphragmatic hernia repair/diaphragm folding
Brain surgery (n = 34, 8.48%)
Implantation of ommaya reservoir
Intracranial haematoma removal
Craniotomy
Meningocele repair
Ventriculoperitoneal shunt surgery
Skull fracture reduction surgery
Others (n = 24, 5.99%)
Sacrococcygeal teratoma resection
Femoral lesion resection
Resection of pharyngeal/laryngeal lesions
Inguinal hernia

### Characteristics and risk factors

Through univariate analysis (Table 2), we found that gestational week (P = 0.003), birth weight (P < 0.001), preoperative hypothermia (P < 0.001), albumin (P < 0.001) and fluid (P = 0.007) infusion, duration of anaesthesia (P < 0.001) and surgery (P < 0.001), application of vasoactive drugs (P = 0.001), magnitude of surgery (P < 0.001), and type of surgery (P < 0.001) were associated with intraoperative hypothermia in neonates. Non-abdominal surgery includes brain surgery, thoracal surgery, and others. Through pairwise analysis, we found that abdominal surgery had a statistically significant difference on intraoperative hypothermia compared with brain surgery (P = 0.003) and others (P < 0.001). In addition, through multivariate logistic regression adjustment analysis (Table 3), we found that gestational week (OR 0.717; 95% CI 0.577–0.890; P = 0.003), preoperative temperature (OR 0.228; 95% CI 0.091–0.571; P = 0.002), duration of anaesthesia (OR 1.052; 95% CI 1.027–1.077; P < 0.001), and type of surgery (OR 2.725; 95% CI 1.292–5.747; P = 0.008) were significantly correlated with intraoperative hypothermia.

**Table 2:** Risk factors associated with intraoperative hypothermia (N = 401)

	Normothermia (n = 76)	Hypothermia (n = 325)	*t*/*χ*^2^/Z	*P*
Age (day) [M(Q)]	7.5 (14)	5 (9)	− 1.940	0.052
Sex (male), n (%)	43 (56.58)	181 (55.69)	0.020	0.899
Weight (kg) (mean ± SD)	3.17 ± 0.58	2.64 ± 0.82	6.502	0.000
Gestational week (weeks) (mean ± SD)	38.21 ± 4.81	36.24 ± 3.78	3.051	0.003
Birth weight (kg) (mean ± SD)	3128.89 ± 521.34	2672.82 ± 845.84	5.879	0.000
Preoperative temperature (℃) [M(Q)]	36.7 (0.3)	36.4 (0.7)	− 5.186	0.000
Duration of surgery (min) [M(Q)]	44 (43)	82 (50)	− 6.972	0.000
Duration of anaesthesia (min) [M(Q)]	85 (55)	134 (57.5)	− 7.850	0.000
Fluid infusion (ml) (mean ± SD)	46.18 ± 28.48	58.39 ± 36.61	− 2.720	0.007
Albumin infusion, n (%)	5 (6.58)	107 (32.92)	21.237	0.000
Blood transfusion, n (%)	5 (6.58)	53 (16.31)	4.712	0.030
Plasma transfusion, n (%)	*3(3.95)*	*38(11.69)*	4.025	0.045
ASA, n (%)			7.957	0.093
I	6 (7.89)	12 (3.69)		
II	54 (71.05)	199 (61.23)		
III	14 (18.42)	103 (31.69)		
IV	2 (2.63)	11 (3.38)		
Type of surgery, n (%)			14.107	0.000
Abdominal surgery	42	249		
Non-abdominal surgery	34	76		
Magnitude of surgery^a^, n (%)			23.286	0.000
Minor surgery	26	38		
Major surgery	50	287		
Invasiveness of surgery, n (%)			0.029	0.854
Endoscopic surgery^b^	18 (23.68)	74 (22.77)		
Non- endoscopic surgery	58 (76.32)	251 (77.23)		
Application of vasoactives, n (%)	4 (5.26)	70 (21.54)	10.842	0.001

P < 0.05 was considered as statistically significant

Data are presented as n (%), mean ± standard deviation or median (interquartile range)

^a^Magnitude of surgery as below: Minor surgery: inguinal hernia; resection of pharyngeal/laryngeal lesions; femoral lesion resection; pyloromyotomy, etc. Major surgery: exploratory laparotomy; ladd’s procedure; tracheoesophageal fistula/oesophageal atresia repair; craniotomy, etc.

^b^Thoracoscope, laparoscopic surgeries

**Table 3:** Multivariable logistic regression results for hypothermia

Variable	OR (95% CI)	P value
Gestational week	0.717 (0.577–0.890)	0.003
Preoperative temperature	0.228 (0.091–0.571)	0.002
Duration of anaesthesia	1.052 (1.027–1.077)	< 0.001
Type of surgery	2.725 (1.292–5.747)	0.008

*OR* odds ratio, *CI* Confidence Interval

P < 0.05 was considered as statistically significant

### Outcomes

Compared to normothermic patients (Table 4), hypothermic patients had longer length of ICU stay (P = 0.001), longer length of hospital stay (P < 0.001), and higher hospital costs (P < 0.001). But there were no association between clinical outcomes and intraoperative hypothermia in the multivariable regression adjusted analysis (Table 5).

**Table 4:** Outcomes of intraoperative hypothermia

	Normothermia (n = 76)	Hypothermia (n = 325)	*t*/*χ*^2^/Z	P
Length of ICU stay[M(Q)] (day)	1 (3)	3 (12)	− 3.334	0.001
Intubation time[M(Q)] (day)	1 (2)	1 (1)	− 0.915	0.360
Length of hospital stay[M(Q)] (day)	12 (16)	19 (21)	− 4.027	0.000
Postoperative bleeding, n (%)	13 (17.11)	85 (26.15)	2.731	0.098
Postoperative pneumonia, n (%)	0 (0)	6 (1.85)	1.424	0.233
Incision infection, n (%)	2 (2.63)	2 (0.62)	2.535	0.111
Deep vein thrombosis, n (%)	0 (0)	3 (0.92)	0.707	0.400
Postoperative death, n (%)	2 (2.63)	20 (6.15)	1.098	0.295
Hospital cost[M(Q)] (RMB)	25,315.85 (19,622.98)	29,916.28 (307,124)	− 3.595	0.000

Data are presented as n (%), median (interquartile range)

P < 0.05 was considered as statistically significant

**Table 5:** Multivariable regression results for outcomes of intraoperative hypothermia

Variable	OR (95% CI)	P value
Postoperative bleeding	1.300 (0.584–2.893)	0.520
Postoperative death	0.587 (0.068–5.059)	0.628

Variable	Unstandardized coefficients	P value
Length of ICU stay	− 3.534	0.247
Length of hospital stay	− 4.086	0.421
Intubation time	− 0.514	0.393
Hospital costs	− 9255.066	0.307

*OR* odds ratio, *CI* Confidence Interval

P < 0.05 was considered as statistically significant

### Intraoperative body temperature curve

**Fig. 2:**
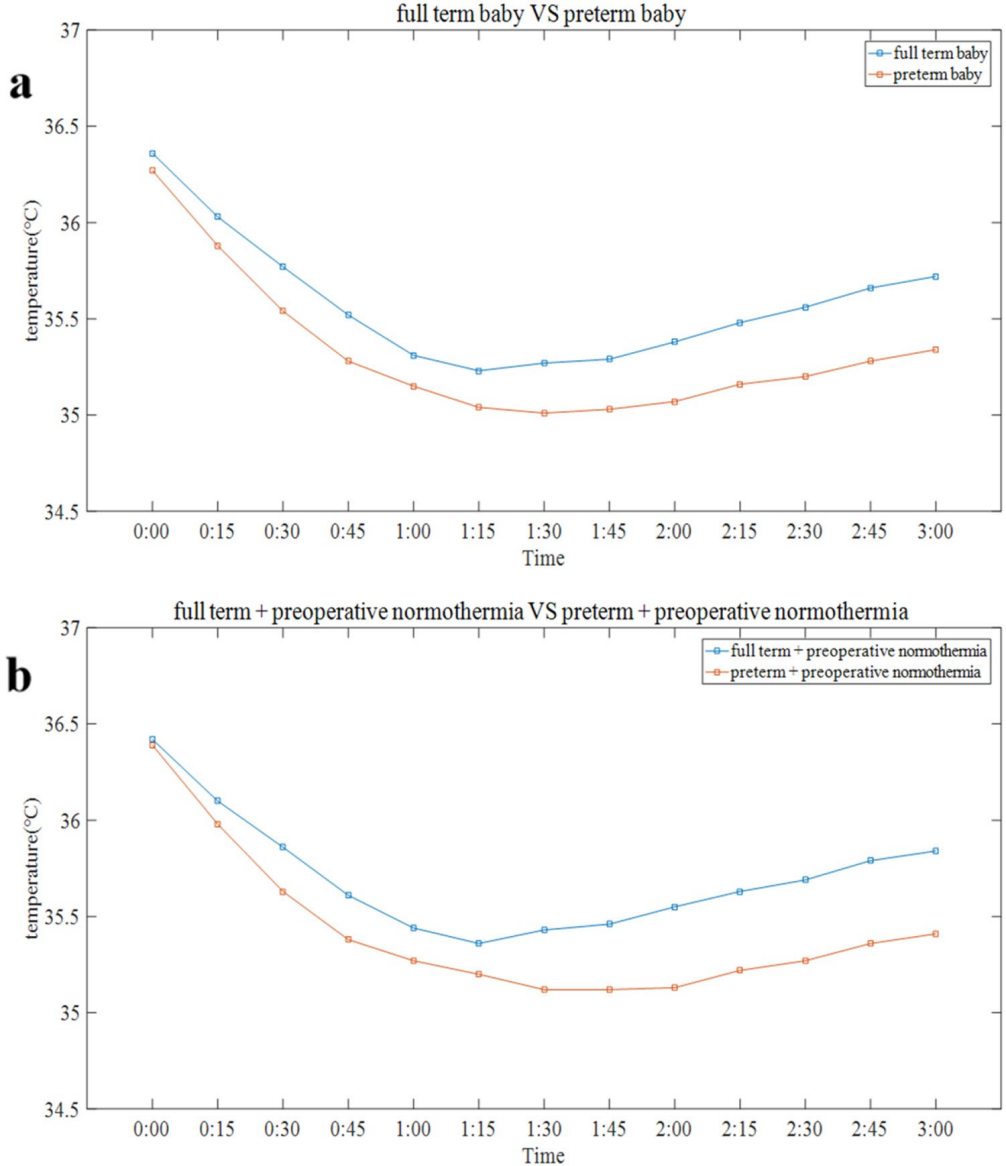
Trends in intraoperative core temperature change in full-term baby and preterm baby. **a** Blue line represents the trend of intraoperative core temperature change in full-term baby; Orange line represents the trend of intraoperative core temperature change in preterm baby. **b** Blue line represents the trend of intraoperative core temperature change in full-term baby with preoperative normothermia; Orange line represents the trend of intraoperative core temperature change in preterm baby with preoperative normothermia

**Fig. 3:**
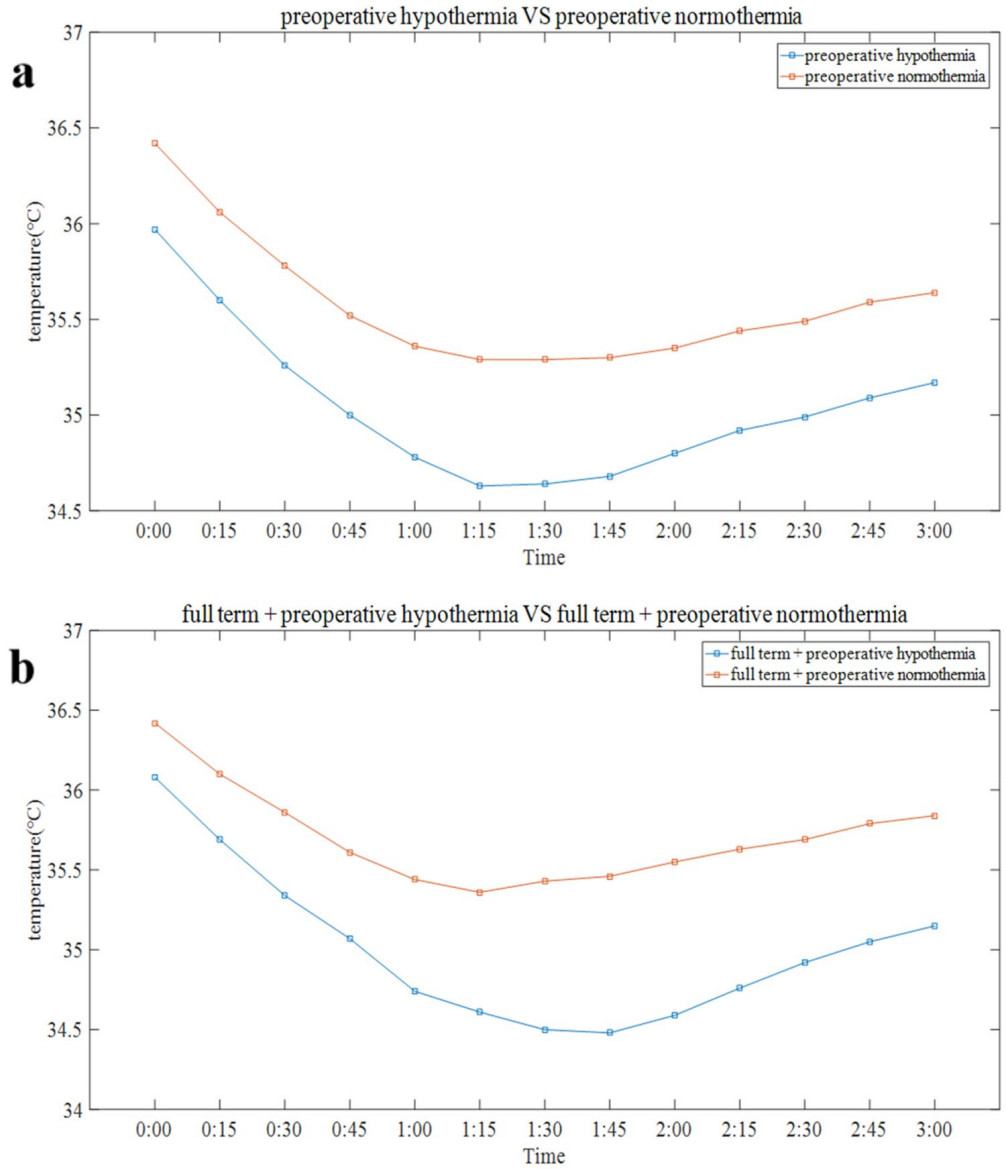
Trends in intraoperative core temperature change in babies with preoperative hypothermia and babies with preoperative normothermia. **a** Blue line represents the trend of intraoperative core temperature change in babies with preoperative normothermia; Orange line represents the trend in intraoperative core temperature change in babies with preoperative hypothermia. **b** Blue line represents the trend of intraoperative core temperature change in full-term baby with preoperative normothermia; Orange line represents the trend of intraoperative core temperature change in full-term baby with preoperative hypothermia

Further analysis of intraoperative body temperature was conducted for the subset of neonates who had undergone an open abdominal surgery procedure and the duration of surgery was more than 2 h (n = 124). For further research, we divided these patients into preterm babies (n = 61) and full-term babies (n = 63), and patients with preoperative hypothermia (n = 28) and patients with preoperative normothermia (n = 96). Figures 2, 3 presents the typical pattern of intraoperative body temperature in neonates. We found that the time for this subset of neonates to reach the lowest point was approximately 1 h 30 min with body temperature decreasing 1–1.5 °C. Then, the body temperature of patients successively entered a short plateau phase and a period of slow ascent. The greatest decrease in core temperatures occurred in preterm babies and neonates with preoperative hypothermia. The lowest intraoperative temperature that occurred in neonates with preoperative hypothermia was lower than 35 °C, which was statistically significant compared with the neonates with preoperative normothermia (P < 0.05). Figures 2, 3

## Discussion

This study shows that there is a high incidence of intraoperative hypothermia in neonates. The intraoperative body temperature curve of neonates is typical. The time for neonates to reach the lowest point was approximately 1 h 30 min. The greatest decrease in core temperatures occurred in preterm babies and neonates with preoperative hypothermia.

Our single-centre study found that 81.05% of neonates experienced intraoperative hypothermia (< 36℃) and 20.45% of neonates experienced intraoperative temperature below 35 ℃. In a national cross-sectional study in China, the results indicated that the overall rate of intraoperative hypothermia was as high as 44.3% in adults [15]. Looking back to previous studies in paediatric patients, the incidence of intraoperative hypothermia was at a high level [16]. In a previous study conducted in Malaysia in 2019, the overall incidence of intraoperative hypothermia was 53.2%, with hypothermia defined as a fall in body temperature to below 35 °C [17]. The percentage of neonates with hypothermia was 83.3%. This study also showed that paediatric patients were unable to maintain core temperature regardless of whether various types of maintenance measures were taken during surgery. There are also existences of high incidence of intraoperative hypothermia in related studies in developed countries [16, 18].Thus, it is important to identify the risk factors for intraoperative hypothermia to carry out optimal and comprehensive thermal management to promote better clinical outcomes of patients.

Our study showed that gestational week is the main risk factor for intraoperative hypothermia among neonatal patients; neonates with fewer gestational weeks are more likely to have intraoperative hypothermia. The intraoperative body temperature of premature babies was lower than that of full-term babies. A previous small study of 88 cases in northern Ethiopia reported that the risk of hypothermia for preterm infants is 3.6 times that of full-term infants [19]. Mortality is also significantly related to lower body temperature and has a clear dose–response relationship in prematurity [20]. Although premature infants regulate temperature better than might be expected [13], the intraoperative temperature of preterm babies recovered relatively slowly after the plateau in our study. Therefore, insulation measures should be taken in neonates, especially preterm infants, to avoid intraoperative hypothermia.

The influence of operation time and anaesthesia time on intraoperative hypothermia has been researched in previous studies [21, 22]. In clinical observation, the duration of anaesthesia in neonatal surgery mainly depends on the time of arterial or venous puncture. Neonatal puncture is very difficult, and the puncture time can be shortened by an experienced anaesthesiologist with the help of ultrasound [23, 24]. Additionally, warmed intravenous fluids seem to make patients warmer during surgery than room temperature fluids, and as a single modality, they can effectively minimize perioperative hypothermia [25, 26]. Although blood product transfusion and fluid transfusion showed no association with intraoperative hypothermia in our research, insulated infusion devices cannot be neglected during neonatal surgery to avoid intraoperative hypothermia.

Lower preoperative temperature is another main risk factor for intraoperative hypothermia among neonatal patients. Neonates with preoperative hypothermia had lower intraoperative body temperature than others with preoperative normothermia. This result reveals the importance of maintaining a suitable preoperative body temperature, including during transportation to the operating room, ICU, emergency room, and even from birth. A previous study proved that the admission hypothermia rate was significantly associated with early neonatal death regardless of hospital performance [27]. It is very important to reduce the incidence of admission hypothermia and increase the median temperature of admission [28]. In a retrospective survey conducted by Bukur et al., 44.6% of subjects presented with prehospital hypothermia among 21,023 trauma patients, and prehospital hypothermia was associated with many adverse consequences [29]. Recent studies show that a key means of reducing perioperative hypothermia is to maintain normal body temperature before transport to the operating room and at other select time points throughout the perioperative period [30]. Our research found that the lowest intraoperative body temperature of neonates with preoperative hypothermia was lower than 35 °C. Therefore, it is necessary to take preheating measures for these patients in addition to conventional insulation measures. The initial rapid reduction in core body temperature after induction of anaesthesia results from redistribution of body heat [12]. A previous study in adults showed that after 1 h of anaesthesia, core temperature decreased 1.6 °C, and redistribution contributed 81% to the decrease [31]. Although redistribution contributes less to the initial postinduction hypothermia in infants than in adults and older children due to the larger fractions of their mass in the torso, it remains the main factor [14]. A prewarming plan before the induction of anaesthesia may play an important role in neonates undergoing surgery under general anaesthesia, especially in neonates with preoperative hypothermia. A previous study proved that a minimum of 30 min of preoperative warming decreased intraoperative hypothermic exposure [32]. Warming patients before induction of anaesthesia does not increase core temperature, but absorbed heat does increase the temperature of peripheral tissues, thus reducing the normal core-to-peripheral tissue temperature gradient [33] and the impact of general anaesthesia. Due to the surgical turnover rate and the condition of the child in clinical practice, it may be difficult to complete the preheating procedures for 30 min. Therefore, we should try to develop a preheating plan to warm up high-risk children with intraoperative hypothermia for a certain period of time when the patient's condition allows for it.

Although the clinical harm of intraoperative hypothermia was lower than expected in our research, the adverse effects of hypothermia cannot be ignored. Previous studies have shown that intraoperative hypothermia was associated with higher risks of clinical adverse events. It is still critical to normalize hypothermia prevention at this stage. Hypothermia is an independent risk factor for mortality and is related to the cardiovascular system, coagulation function, acid–base balance disorder, and respiratory distress [34]. Neonates are the most sensitive to the side effects of hypothermia during surgery. Hypothermia will impair platelet function, the activity of clotting factors, and fibrinogen synthesis [35]. Coagulation dysfunction increases the amount of bleeding, thereby increasing the rate of blood transfusion, and the incidence of surgical infections is higher. In addition to the effect on blood coagulation, cold stress can lead to metabolic acidosis, hypoglycaemia, and other consequences. In addition, the effects of hypothermia on the pharmacokinetics of anaesthesia include reducing the minimum alveolar concentration of inhaled anaesthetics, prolonging the effect of most nondepolarizing muscle relaxants, and prolonging the half-life of drugs excreted by the liver and kidneys [36, 37]. Our study showed that the lowest body temperature that occurred in neonates with preoperative hypothermia was lower than 35 °C. Therefore, comprehensive insulation measures should be taken in this set of neonates. We perform the preheating plan mentioned above when conditions permit. The time for neonates to reach the lowest point is approximately 1 h 30 min, which is much faster than that for adults. This result is of clinical significance. We should take insulation measures as soon as possible and give priority to high-risk children in the case of insufficient insulation equipment to reduce the impact of hypothermia.

Like most observational studies, our research has some limitations. First, this study is a single-centre study, and the cases have certain limitations. It is necessary to conduct multicentre, large-scale clinical trials for verification. Second, standardized procedures for heat preservation of neonates were not strictly followed, and there was no strict reference standard for the judgement of postoperative complications. It is necessary to conduct prospective studies to further verify this hypothesis.

## Conclusion

This study shows that there is a high incidence of intraoperative hypothermia in the neonate population. We should take comprehensive insulation measures as soon as possible and give priority to high-risk children in the case of insufficient insulation equipment. The findings of our study may contribute to the limited literature on intraoperative hypothermia in neonates and better guide the application of insulation measures in the operating room. This is particularly important in developing countries where insulation equipment is insufficient.

## Data Availability

The datasets generated and/or analysed during the current study are available from the corresponding author upon reasonable request.

## Abbreviations

ASA: American Society of Anaesthesiologists
ICU: Intensive care unit
SpO_2_: Haemoglobin oxygen saturation
NIBP: Noninvasive blood pressure
IBP: Invasive blood pressure
MAC: Minimum alveolar concentration
ECG: Electrocardiogram
